# Factors and co-factors influencing clinical manifestations in nsLTPs allergy: between the good and the bad

**DOI:** 10.3389/falgy.2023.1253304

**Published:** 2023-09-28

**Authors:** Erminia Ridolo, Alessandro Barone, Martina Ottoni, Silvia Peveri, Marcello Montagni, Francesca Nicoletta

**Affiliations:** ^1^ Department of Medicine and Surgery, University of Parma, Parma, Italy; ^2^ Allergology Unit, University Hospital of Parma, Parma, Italy; ^3^ Departmental Unit of Allergology, Guglielmo da Saliceto Hospital, Piacenza, Italy

**Keywords:** anaphylaxis, co-factor, cross-reaction, food allergy, food sensitization, lipid transfer protein

## Abstract

Non-specific lipid transfer proteins (nsLTPs) are a family of plant pan-allergens that represent the primary cause of food allergies in the Mediterranean area, characterized by a wide range of clinical manifestations, ranging from the total absence of symptoms up to anaphylaxis. This wide variety of symptoms is related to the intrinsic capacity of nsLTPs to cause an allergic reaction in a specific subject, but also to the presence of co-factors exacerbating (i.e., exercise, NSAIDs, PPIs, alcohol, cannabis, prolonged fasting, menstruation, acute infections, sleep deprivation, chronic urticaria) or protecting from (i.e., co-sensitization to PR10, profilin or polcalcin) severe reactions. In this picture, recognizing some nsLTPs-related peculiarities (i.e., route, type and number of sensitizations, concentration of the allergen, cross-reactions) and eventual co-factors may help the allergist to define the risk profile of the single patient, in order to promote the appropriate management of the allergy from dietary advices up to the prescription of life-saving epinephrine autoinjector.

## 1. Introduction

Non-specific lipid transfer proteins (nsLTPs), firstly described as fruit allergens in 1999, constitute a family of plant pan-allergens characterized by their resistance to heat and to gastrointestinal digestion (1). nsLTPs sensitization is mostly reported in the southern countries of Europe, such as Italy and Spain, where it represents an “endemic” cause of primary food allergy and where the principle responsible is peach-LTP (2). Particularly, in the south of Italy the prevalence of nsLTPs sensitization is 27.2%, with a gradual decrease heading north, and almost the absence of such a sensitization in the north of the Alps. In the rest of Europe, nsLTPs sensitization does not represent a frequent cause of food allergy, highlighting the strong relationship between this type of allergy and a Mediterranean climate, rather than a continental one (3). In this scenario, a primary sensitization to pollen-LTPs is thought to play a relevant role in northern hemisphere (4, 5).

More precisely, in the context of nsLTPs sensitization, a wide range of clinical expressions have been described, extending from the total absence of symptoms, through mild manifestations, such as oral allergic syndrome or contact urticaria, up to systemic ones, involving cutaneous (with urticaria and angioedema), respiratory (with rhino-conjunctivitis, asthma and bronchospasm) and gastrointestinal (with nausea, vomiting, diarrhoea) systems, and worsening to anaphylaxis. nsLTPs allergy, in fact, represents the most important cause of food-induced anaphylaxis in the Mediterranean area (5). Furthermore, it is already well known that IgE reactivity to nsLTPs may often require several co-factors to become clinically exacerbated (6).

A wide variety of elements determines the clinical manifestation of nsLTPs sensitization, including both the intrinsic characteristics of nsLTPs to sensitize and promote the development of the allergy in a specific subject, and concomitant factors (co-factors) someway protecting from or contributing to the outbreak of the allergic reaction. The aim of this review is to make a concise and orderly overview about the factors and co-factors currently known. This may help to predict the eventual clinical manifestations subsequent to nsLTPs sensitization, as well as their severity, providing the allergist a greater clarity, and leading to an easier management also of the more undefined cases of nsLTPs allergy.

## 2. Structural features of nsLTPs and primary sensitization

At the moment, within the huge family of nsLTPs, the allergens recognized from edible parts of plant-foods are classifiable in two families based on their molecular weight: nsLTP1s (>9 kDa), comprising the large part of the nsLTPs responsible for the development of allergic reactions, and nsLTP2s (<9 kDa) (2, 7).

The pivotal role played by peach-LTP (*Prunus persica*), Pru p 3, as primary sensitizer by crossing the intestinal monolayer and inducing the production of T helper 2 cytokines because of several T-cell activating regions is widely reported in literature (8–10). On the other hand, nsLTPs from less related species, such as Cor a 8 in hazelnut (*Corylus avellane*) and Hel a 3 in sunflower seed (*Helianthus annuus*), do not share these T-cell epitope sequences, suggesting for them a lower probability to act as primary sensitizer (2, 11), particularly for hazelnut-LTP for which no major T-cell-activating region was found (11). Moreover, four main epitopes of Pru p 3 recognized by serum specific IgE have been described as shared among the members of the *Rosaceae* family, predisposing the patient to eventual cross-reactions, even after the ingestion of plant-foods different from peach but nsLTPs containing (12, 13).

The resistance of nsLTPs to high temperature and pepsin digestion is mainly due to the presence of disulphide bridges in their structure, probably with a different selective stability among the different nsLTPs. A cleavage of these bonds has been reported through *in vitro* experiments for Pru p 3 and Cor a 8 conducted at neutral pH and high temperature (14, 15). Moreover, Cor a 8 is more rapidly degraded by lysosomal proteases than Pru p 3 (13). Maize-LTP (*Zea mays*), Zea m 14, instead, has been found to elicit unchanged binding capacity to specific IgE from patients with anaphylaxis following corn ingestion or positive double-blind placebo-controlled food challenges, even after cooking to 100°C (16).

## 3. Capacity of nsLTPs to cause allergic reactions

Lots of factors may affect the capacity of nsLTPs to cause clinical manifestations, particularly the type of primary sensitizer, the route of sensitization, the concentration of nsLTPs in the culprit foods, the cross-reactivity, the patient's age, and the geographic area (2).

Following, the discussion of the key aspects in the onset of allergic reactions to nsLTPs.

### 3.1. Route of sensitization

As for many other food allergies, the gastrointestinal tract is thought to be a common route of sensitization also for nsLTPs, and thanks to its capacity to resist the gastric environment, Pru p 3 shows itself as the most important primary sensitizer (8, 9). Aside from the gastrointestinal sensitization, since in some cases the determination of the source of sensitization is not possible, even other more infrequent routes have always to be kept in mind during the anamnesis, in order not to neglect possible further causes of reaction, different from the ones already alleged.

For what concerns the cutaneous route, contact urticaria induced by peach peel was significantly more frequent in patients hypersensitive to Pru p 3 than in subjects with pollen-food allergy syndrome related to Pru p 1 or Pru p 4 (63% vs. 6%), and in several cases, contact urticaria by Pru p 3 has been described as preceding the onset of the food allergy by years (17). Furthermore, nsLTPs hypersensitivity is often already present in early life, especially in children with atopic eczema, and may precede the first ingestion of several foods (3). Recently, a murine model supported the hypothesis of a skin-mediated allergic sensitization: the exposure of Pru p 3 on depilated skin favored the occurrence of anaphylaxis after intraperitoneal provocation, and promoted CD45+ infiltration, also across other tissues, such as mucosa, lungs, and gut, validating the hypothesis of a systemization of the response (18).

Furthermore, the respiratory system may offer another route of sensitization, as exemplified in several cases of occupational respiratory allergy experienced by peach, asparagus, wheat and maize crop workers (16, 19–21), causing both respiratory symptoms and also severe reactions after the ingestion of nsLTPs-containing plant-foods (22). nsLTPs, in fact, play a defensive role, especially in the most exposed surfaces of the plants, therefore their expression may be increased in crops affected by both biotic (i.e., fungal infections due to *Ustilago maydis* or *Fusarium graminearum*) or abiotic stresses (i.e., drought, cold, and salt), favouring higher exposure to these allergens (23, 24). Another example of nsLTPs occupational allergy is baker's asthma, due to the primary sensitization to flour caused by inhalation of Tri a 14 in wheat (*Triticum aestivum*) (25). Even in this case, murine models, intranasally sensitized with Pru p3 combined with lipopolysaccharide as adjuvant, support the respiratory system as a route of sensitization, showing a T helper 2 response and anaphylactic symptoms after intraperitoneal provocation (26). Still in the context of the respiratory route, less investigated causes of sensitization have also to be considered, such as the inhalation both active and passive of Can s 3 (LTP of *Cannabis sativa*) from the smoke of marijuana due to the increasing social, medical and occupational exposure (3, 27). In addition, the cutaneous exposure or the ingestion of cannabis, for example through cake, tea, oil or seeds, in some cases might be suspected to play as a route of sensitization, but it is necessary to specify that no unquestionable certainties have been provided (27, 28). Can s 3 may trigger a variety of symptoms from mild ones, such as contact urticaria or rhino-conjunctivitis, to more important clinical reactions, like life-threatening anaphylaxis (28–30). In addition, a primary Can s 3 sensitization may favour the onset of plant-foods allergies even in the absence of Pru p 3 sensitization, whose amino acid identity is 64% (30). Symptoms reported with plant-foods ingestion after cannabis exposure occur often with food sources different from the ones usually seen in the Bet v1-related pollen-food syndrome, such as banana, tomato, citrus and grapefruits, and are often described as more severe and systemic, thus suggesting a correlation with nsLTPs allergy (28).

### 3.2. Concentration of nsLTPs in the different culprit foods

Considering that a higher amount of the allergen could be related to a greater exposure to such protein, the differences in nsLTPs concentrations among plant-foods of different families, among diverse species of the same family, and even among distinct parts of the same fruit might influence eventual sensitizations or allergic reactions. The data concerning the quantification of nsLTPs for the different plant-foods, tough, are still fragmentary in literature.

Regarding the *Rosaceae* family, the greater concentration of nsLTPs is contained in the peel, but not in a similar concentration for all the members: while the concentration of Pru p 3 in peach peel is approximately 6 mg/g tissue, Mal d 3 in apple (*Malus domestica*) peel is consistently lower (approximately 66 μg/g tissue) (11, 31). Notably, as opposed to another severe peach allergen, the gibberellin-regulated protein, a Japanese study reported a concentration of Pru p 3 in the pulp of different varieties of peach much lower than in the peel, ranging from 0.1 to 12.0 μg/g tissue (32). As a consequence of these results, it should be a good practice to recommend the patients sensitized to nsLTPs always to peel the fruits before the ingestion, particularly those of the *Rosaceae* family. Moreover, a relevant amount of Pru p 3 is contained also in peach leaves (approximately 0.8 μg/g tissue) (32), supporting additionally the idea of a possible role in the sensitization through less common routes (i.e., cutaneous or respiratory) in subjects more exposed, as for example in crop workers. Interestingly, traces of Pru p 3 (maximal concentration of 0.03 μg/g lotion) have been detected also in several cosmetic lotions (32), highlighting potential hidden causes, albeit in rare circumstances, of allergic reactions.

Regarding nuts, Jug r 3 of walnut (*Juglans regia*), as Cor a 8 of hazelnut, is contained mainly in the brown skin of the embryo, with a concentration per nut comparatively lower than peach (33). In peanut (*Arachis hypogaea*), the concentration of LTP (Ara h 9) related to the amount of all peanut allergens (<6.5 pmol/mg) is considerably lower than the concentration of seed storage proteins Ara h 1, Ara h2, Ara h 3 and Ara h 6 (34).

Zea m 14 concentration in maize kernels has also been determined, ranging from 29 to 1,030 μg/g tissue in 14 different lines (35). As already described above, such a wide range may reflect for example the expression of different levels of nsLTPs in response to the presence or the absence of eventual biotic or abiotic stresses, this making possible to speculate that the exposure to different concentrations of the allergen might contribute to the variety in the nsLTPs allergic manifestations.

### 3.3. Capacity of nsLTPs to induce clinical cross-reactivity

The degree of sequence homology in nsLTPs from vegetable species ranges from 35% to 95% and this plays a key role in the context of immunological cross-reactivity caused by foods different from the ones involved in former allergic reactions (36). Considering sera from patients with nsLTPs syndrome, which is characterized by a spectrum of allergic manifestations to multiple nsLTPs, the IgE capacity to bind different purified nsLTPs is most frequently reported for Pru p 3 (peach), followed by Mal d 3 (apple), Cit r 3 (*Citrus reticulata*; orange), Bra o 3 (*Brassica oleracea*; cabbage), Sin a 3 (*Sinapis alba*; mustard), Jug r 3 (walnut) and Cas s 8 (*Castanea sativa*; chestnut). The wheat-LTP, Tri a 14, shows a less strong relationship with other nsLTPs, suggesting a different pattern of recognition (37, 38): despite a sequence identity of 45% between Tri a 14 and Pru p 3, the low clinical cross-reactivity detected may reflect the differences in their tridimensional IgE-binding regions (14).

Moreover, the cross-sensitization to nsLTPs from other sources other than peach-LTP follows a hierarchical order in nsLTPs syndrome (apple at second place, followed by nuts and, much less frequently, cereals, cabbage, mustard, beer, lettuce and other foods) that may also be influenced by the degree of homology to Pru p 3 (3, 38–41). Nevertheless cross-sensitization does not necessarily mean cross-reactivity. In fact, despite the evidence of relevant levels of specific IgE for nsLTPs of some foods other than peach (like lentil, soybean and maize), almost no clinical reactions have been reported after their ingestion (39).

As already mentioned above, inhalation is a route of sensitization to nsLTPs. Considering the homology of sequence with Pru p 3, pollen-LTPs can be divided in two subsets, including mugwort (*Artemisia vulgaris*) Art v 3 and plane tree (*Platanus acerifolia*) Pla a 3 on a side (41% and 46% of sequence identity respectively), and pellitory (*Parietaria judaica*) Par j 1 and Par j 2, ragweed (*Ambrosia artemisifolia*) Amb a 6 and olive tree (*Olea europaea*) Ole e 7 on the other side (with corresponding sequence identities below 30%) (35, 39); no cypress-LTP is currently known (42). Cases mediated by a primary sensitization to Art v 3 and Pla a 3 have been described particularly in northern Europe (43), as causing a pollen-food syndrome, triggering both respiratory symptoms, such as rhino-conjunctivitis, and food reactions to nsLTPs. In this regard, mugwort and plane tree pollen extracts have led to an inhibition between 50% and 100% of IgE specific to nsLTPs containing foods in microarray (38). This is worthy, especially in those areas with high exposure to Art v 3 and Pla a 3, as respectively China and Gran Canaria (2). Recent Italian follow up data, though, point against a primary pollen LTP sensitization, and rather support this occurrence as a consequence of cross-reactions to other primary sensitizers. Particularly, in this study baseline Pru p 3 IgE levels exceeded Art v 3 IgE levels in 84% of those sensitized to both allergens, and the mean specific levels of IgE to Art v 3 and Pla a 3 increased significantly in the presence of Pru p 3 reactivity, unlike the case of the IgE to Ole e 7. Moreover, the absorption of sera from three patients sensitized to Art v 3, Pla a 3 and Pru p 3 with commercial extracts of *Artemisia vulgaris* and *Platanus acerifolia* in no case induced a relevant inhibition (>75%) of IgE reactivity to Pru p 3, unlike for the IgE reactivity to Art v 3 and Pla a 3. This indirectly suggests that neither planetree nor mugwort act as primary sensitizers in patients with nsLTPs allergy (42).

Furthermore, the cases of sensitization to wall pellitory Par j 2 in areas where those plants do not naturally grow, like in northern Europe, represents another evidence of cross-reaction among different nsLTPs (43). Compared to Pla a 3 and Art v 3, sensitization to Par j 2 has been associated to a significant lower rate of food induced systemic reactions but a higher prevalence of bronchial asthma (38).

### 3.4. Level of specific IgE to nsLTPs

Pru p 3 has been recognized as the most frequently occurring allergen responsible for nsLTP sensitization, especially in sensitized to multiple nsLTPs (44) and at younger age, finding also that Jug r 3 recognition may be comparable in patients older than 15 years (38). Despite the level of specific IgE to Pru p 3 production is higher at younger age (45), the risk of anaphylaxis related to Pru p 3 sensitization is lower in patients aged 25 years or younger (46).

In addition, the evidence of sensitization to more than 5 nsLTP molecules is significantly linked with an increased risk of systemic reactions (38), as confirmed by another study where the presence of IgE to 4 or more nsLTPs was associated to reactions more severe, with an increasing of the probability for any further nsLTP sensitization (44).

Regarding the correlation between the level of specific IgE to nsLTPs and the severity of the allergic reactions, there are discordant results and no certainties. To our knowledge, no significant correlation has been reported (44), but on the other hand, higher ISU-E values have been associated to systemic reactions considering measurements executed with ISAC microarray (a semi-quantitative assay) (38).

## 4. The role of the co-factors in allergic reactions to nsLTPs

Besides the inherent capacity of nsLTPs to trigger allergic reactions, other precipitating or protective co-factors need to be considered. To this end, here is the discussion of the main and more deepened topics.

### 4.1. Co-sensitizations

Patients who were simultaneously sensitized to nsLTPs, PR-10 and profilins reported a higher prevalence of local symptoms to foods (oral allergic syndrome) and a lower prevalence of systemic symptoms (7, 38). A significant lower risk of anaphylaxis has been reported also in patients with a concomitant sensitization to nsLTPs and polcalcin Bet v 4 (46). A possible explanation to this “protective” effect mediated by pollen panallergens may be the partial occupancy of FceRI on mast cells and basophils by specific IgE other than the ones specific for nsLTPs, reducing in this way the chance of cross-linking and consequently degranulation and histamine release (47).

As for co-sensitization to nsLTPs and cross-reactive carbohydrate determinants (CCD), no clinical correlations were observed (38).

Leaving aside the co-sensitization with panallergens from pollen, a retrospective single-center study has recently evaluated the clinical implications of the co-sensitization to nickel in patients sensitized to nsLTPs. Surprisingly, fewer cutaneous symptoms, in particular contact-urticaria, have been reported in patients with systemic nickel sulfate allergy syndrome (48), but no clear explanation of this finding has been currently provided in literature.

### 4.2. Co-factors precipitating reactions to nsLTPS

Unlike other stable food allergens, the clinical exacerbation of the reaction after the ingestion of a nsLTPs containing food otherwise tolerated, as well its worsening, is often related to the presence of at least one co-factor (6). A recent study of Ruano-Zaragoza et al. conducted on a cohort of 528 patients allergic to Pru p 3 highlighted that 37% of the patients reported allergic reactions in presence of at least one co-factor among physical exercise, NSAIDs (non-steroidal anti-inflammatory drugs), alcohol, menstruation or sleep deprivation, and experienced more severe reactions then those in absence of such co-factors, with a later onset (27 vs. 16 years). Regarding the foods involved, *Rosaceae* and lettuce, as well as the ingestion of a mix of foods containing nsLTPs in the same meal, had a significant relationship with the reactions of the co-factor group. Exercise and NSAIDs were the most reported co-factors (41.7% and 37.8% respectively) (49). Some of these observations confirm the results reported in an Italian study that assessed the effect also of other co-factors in patients sensitized to Pru p 3 and Tri a 14, confirming a later onset of the co-factors related reactions in adulthood rather than childhood. Also in this case, in fact, exercise was the most common co-factor reported (31.9%), followed by humid-heat in 31.2%, pollen peak in 17.8%, NSAIDs in 15.9%, alcohol in 9.6%, and PPI (pump proton inhibitors) in 4.5% (50).

#### 4.2.1. Physical exercise

The importance of the exercise as a co-factor is so relevant as to be related to a specific nosological entity, that is Food-Dependent Exercise-Induced Anaphylaxis (FDEIA), a condition whereby the onset of anaphylaxis occurs during or soon after physical exercise preceded by ingestion of a food, while the food and exercise are tolerated separately (51). Some of these patients experience FDEIA after all meals followed by exercise, regardless of the food eaten, while in other cases the reaction occurs only in presence of specific allergens (52). In Italy nsLTPs represent the main allergens involved in such a category of anaphylaxis (3, 52), but other food allergens might be involved (48). The pathological mechanism is probably due to the increase of the allergen absorption caused by the elevated blood circulation, with the subsequent increased transport of the allergen by blood flow redistribution during exercise from gut to tissues containing mast cells (51, 53). Moreover, increased plasma noradrenaline levels might induce anaphylactoid manifestations (51).

Particularly, FDEIA is often associated to wheat consumption and, in those cases, it is named WDEIA (wheat-dependent exercise-induced anaphylaxis) (52, 53). Some evidence suggests that Tri tu 14, LTP of *Triticum turgidum* (used for pasta, pizza, bulgur, semolina and couscous), rather than Tri a 14 is associated with WDEIA, as well as with Pru p 3-mediated food allergy (16). Additional co-factors reported as facilitator for WDEIA are both menstruation, for which the pathological mechanism is not yet fully understood (52, 53), and cannabis consumption (55), probably due to the cytokine release mediated by the stimulation of CB2 receptors on the immune cells by cannabinoids (56). Even though there are no reports about the specific effect on human mast cells, it may be relevant to state also that cannabidiol, which is another component of cannabis, promotes a Ca2+ dependent activation of a rat basophil leukaemia mast cell line, alone or together with Fc*ε*RI stimulation (57).

#### 4.2.2. Non-steroidal anti-inflammatory drugs

Among the best characterized co-factors there are the concomitant consumption of NSAIDs, able to enhance IgE mediated activation of basophils (6, 58). Moreover, NSAIDs-intolerance, as well as chronic urticaria, are clinical conditions that make mast cells more easily excitable by allergens (59). Notably, food-dependent NSAIDs-induced hypersensitivity reactions (FDNIH) are in a relevant number of cases related to a clinical history of NSAIDs hypersensitivity, with Pru p 3 described as risk factor, this suggesting the evaluation of nsLTPs sensitization in the diagnostic workup for these patients (60).

Furthermore, it is worth recalling that NSAIDs intake to control menstrual pain, as well as during acute infections, is a frequent practice, determining the risky concomitance of multiple co-factors (50).

#### 4.2.3. Alcohol

Alcohol consumption has been related to nsLTPs anaphylaxis, but little is known about its mechanism. A possible explanation involves the increasing of the allergen absorption related to the relaxing of the tight junctions in gut epithelium (61). Hypothetically, it may also act as an augmenting factor because of the ingredients in alcoholic beverages (i.e., sulphites) (62), not to mention eventual reactions to LTPs of both beer and wine eventually not fully investigated (63, 64). In nsLTPs related FDEIA, alcohol has been reported as a co-factor in lower percentage than in FDEIA omega 5 gliadine-related, but however with a relevant frequency (12.2% vs. 55.6% respectively), nearly confirming the previous data reported in European registries (up to 15.6%) (61).

#### 4.2.4. Less investigated causes

Even a prolonged fasting may act as a risk factor for anaphylaxis, especially in case of the isolated ingestion of the offending food, probably because an empty gastrointestinal tract may lead to a more rapid absorption, impeding pepsin to break down the allergen matrix more efficiently (6, 59). For the same reason, the use of proton pump inhibitors may promote a higher absorption of nsLTPs (65).

Additionally, several trials count sleep deprivation among the possible risk factors in nsLTPs-related reactions (49, 66), but no additional comments are provided at this regard. Even though the exclusion of patients monosensitized to Ara h 9, it might be interesting to state as a cue that a randomized trial performing a blinded challenge tests in peanut-allergic patients described also sleep deprivation as a co-factor. More specifically, it has been described to reduce the threshold dose triggering allergic symptoms, thus suggesting a certain creditability of the sleep loss as a promoting factor (67).

Even though the absence of data concerning a specific correlation to nsLTPs to our knowledge, it is well known that, also acute infections lower the threshold of oral tolerance to eventual allergenic foods, probably due to the increased blood circulation. Moreover, gastrointestinal infections might favour the absorption of undigested protein by promoting the inflammation of the mucosa (53).

## 5. Critical points for diagnostic workup and management of the patients with nsLTPs sensitization/allergy

The wide clinical spectrum in nsLTPs sensitization ranges from the total absence of symptoms (i.e., in case of accidental detection due to previous inappropriate prescription of tests in absence of suggestive symptoms, or in case of too extensive screening tests), up to nsLTPs syndrome, characterized by the occurrence of anaphylaxis in the worst cases. In consideration of this, a balanced management of these cases, avoiding both unnecessary alarmism and hyper-permissive approach, results pivotal in ensuring a good quality of life to the patient. At the same time, it represents a very tough challenge for the allergist. In this context, a detailed anamnesis should be never underestimated, with a particular emphasis placed on the offending foods, the clinical manifestations of the allergic reactions (severity of the symptoms, time of onset, recourse to hospitalization and/or adrenaline) and the presence of eventual co-factors. Due to a widespread but often clinically irrelevant IgE cross-reactivity for different type of nsLTPs, during the diagnostic phase an accurate exclusion or confirmation of other responsible food allergens is recommended. A broad screening without clinical necessity, instead, is absolutely discouraged (68) in order to avoid unnecessary dietary restrictions.

**Figure 1:**
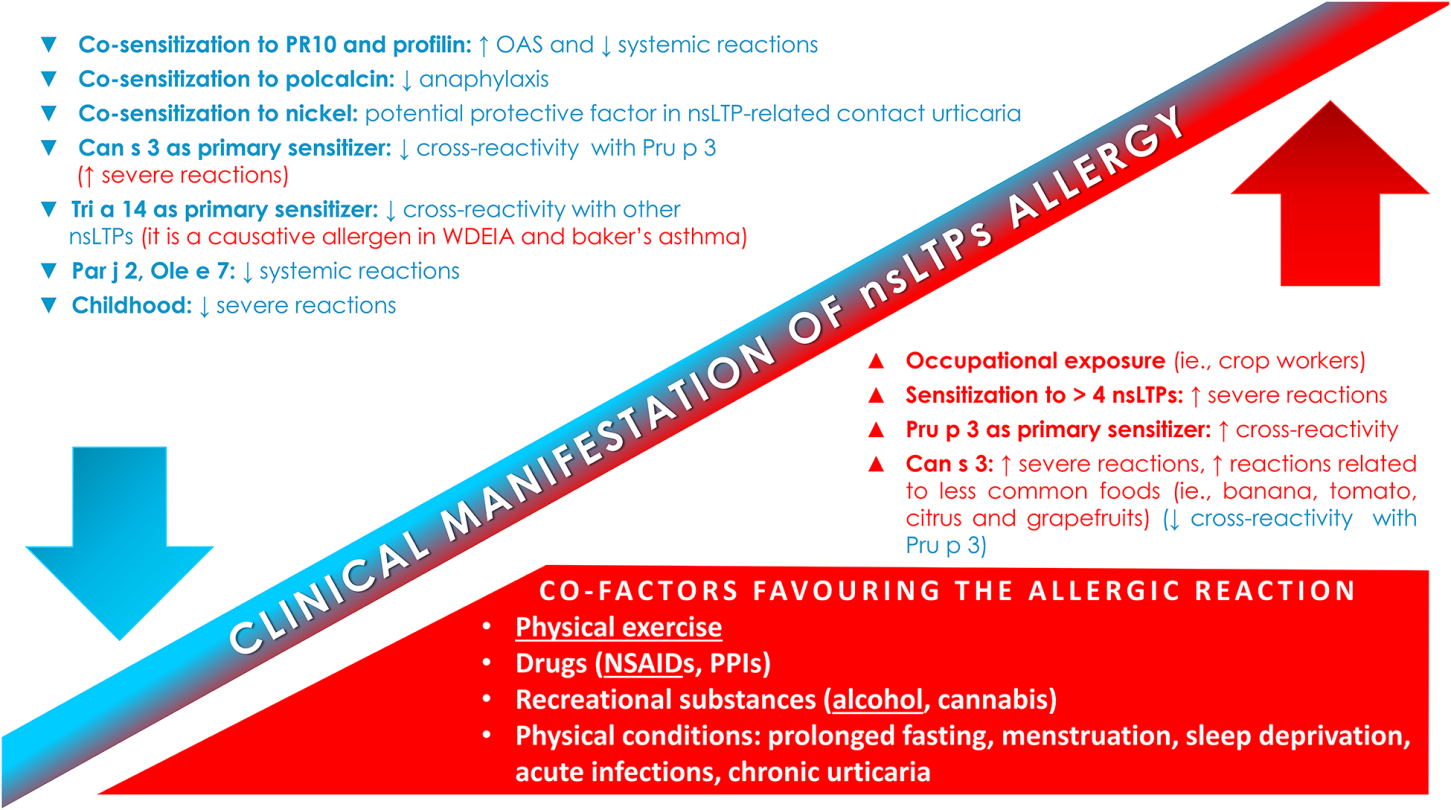
Factors and co-factors influencing the clinical manifestation of nsLTPs allergy. NSAIDs-induced hypersensitivity; NSAIDs, non-steroidal anti-inflammatory drugs; nsLTPs, non-specific lipid transfer protein; OAS, oral allergic syndrome; PPIs, proton pump inhibitors.

Taking into account what already stated above, Figure 1 reports the factors and co-factors influencing the clinical manifestations of allergic reactions to nsLTPs, whereas Supplementary Table S1 resumes several critical points of this topic, proposing at the end an eventual approach, as much as possible personalized, to the management of these patients.

## 6. Conclusion

Given the wide variety of clinical manifestations in nsLTPs allergy, patients affected require a personalized management based on a careful assessment of the individual risk profile. It is necessary to take into account all those factors that may promote or precipitate a reaction, comprising background factors, such as the geographical area, the eventual sensitization to multiple nsLTPs or the occupational exposure (i.e., peach pickers), and concomitant transitory factors. Regarding the latter, physical exercise is the most reported co-factor in reactions to nsLTPs, but there are also other relevant transient co-factors, such as the intake of drugs (i.e., NSAIDs and PPIs) or recreational substances (i.e., alcohol and cannabis), as well as a particular physical state (i.e., prolonged fasting, acute infection, menstruation, sleep deprivation). On the other hand, also eventual factors that may play as protector from severe reactions, such as co-sensitization to PR10, profilin, or polcalcin must be considered. Patients affected by nsLTPs allergy must not be managed all in a standard way: they deserve, instead, a personalized approach, based on their own unique risk profile and ranging from simple dietary advice, up to the prescription of a life-saving device (i.e., the epinephrine autoinjector), aiming to the optimal balance between an unnecessarily hyper-restrictive approach and an excessively permissive one. In this prospective, allergen specific immunotherapy (69), although not yet a routinary practice, might represent a relevant therapeutic option for the future, by the mean of further studies aimed to provide specific biomarkers of safety and effectiveness, as well as standardised protocols (70).

Certainly, much has still to be discovered, particularly about the role of co-factors in anaphylaxis nsLTPs-related, thus also providing important cues for future studies.
