# Supplementary material for: Factors and co-factors influencing clinical manifestations in nsLTPs allergy: between the good and the bad
Source: Front Allergy. 2023 Sep 28;4:1253304. doi: 10.3389/falgy.2023.1253304 (PMC10568476; doi:10.3389/falgy.2023.1253304)
Supplement: Supplementary file 1 [file Table1.docx]

| **Accurate anamnesis through targeted questions** | | | | | | | | | | |
| --- | --- | --- | --- | --- | --- | --- | --- | --- | --- | --- |
| **Source of the offending allergen** | **Clinical manifestations of the allergic reaction** | | **Route of sensitization** | | **Geographical**  **Area** | **Occupational risk** | | **Co-sensitizations** | | **Eliciting/augmenting co-factors** |
|  | | | | | | | | | | |
| Suspected food raw/cooked, peeled/unpeeled.  Often a hierarchical sequence in nsLTPs syndrome: *Rosaceae*, followed by nuts and less frequently cereals and other foods. (1)  Banana, tomato, citrus and grapefruits more frequent in cannabis-related reactions. (2) | Time of onset: later onset in CFDR than in non-dependent (90 vs 45 min). (3)  Symptoms: local (OAS, contact urticaria, angioedema); systemic (involving cutaneous, respiratory, gastrointestinal and/or vascular districts). (4)  Greater severity in CFDR. (5)  Age of onset: more likely in adulthood in CFDR. (6) | | Gastrointestinal (more frequent). (7, 8)  Cutaneous (less frequent). Contact urticaria by Pru p 3 might precede the onset of the FA by years. (9)  Respiratory (less frequent). Consider cannabis smoke, (10, 11) plane tree and mugwort pollens. (12) | | nsLTPs FA is much more frequent in the Mediterranean area. (13, 14) | Crop workers (15)  Bakers (investigate wheat-LTP). (16) | | Differential diagnosis  Concomitant sensitization to PR-10, profilins and polcalcin are related to a lower risk of anaphylaxis. (17, 18, 19)  Concomitant sensitization to nickel is related to lower contact-urticaria by nsLTPs (20). | | Physical exercise is the most frequent: investigate FDEIA, particularly WDEIA. (21, 22)  Drugs: NSAIDs (investigate also eventual NSAIDs hypersensitivity) (23); PPIs. (24)  Recreational substances: alcohol, (25) cannabis. (26)  Physical conditions: menstruation, (27) prolonged fasting, (27) acute infections, (22) sleep deprivation, (5, 28) chronic urticaria. (27) |
| **Diagnostic tests** | | | | | | | | | | |
| **A broad screening without clinical necessity is discouraged.** (29) | | **Accurate exclusion or confirmation of other suspected food allergies is recommended.** (29) | | I line test:  **Skin prick tests (SPTs)** by employing commercial extracts of the suspected food.  **Prick by prick test (PPTs)** using the unpeeled fresh suspected fruit adds diagnostic value. (29) In the case of cannabis, if possible, crude cannabis products (leaves,seeds, and buds) should be used. (30) | | | II line test:  **Serum specific IgE antibodies (sIgE)** might further foster the results of SPTs and PPTs.  **Component resolved diagnosis (CRD)** allows achieving a greater specificity, by using single molecular allergenic components. (29) | | **Oral food challenge in a controlled setting:** the only diagnostic test able to define clearly if a plant-food might be tolerated, avoiding unnecessary dietary limitations. (29) | |
|  | | | | | | | | | | |
| Due to a widespread but often clinically irrelevant IgE cross-reactivity for different type of nsLTPs.  To avoid unnecessary dietary restrictions. | |  | | Since Pru p 3 covers a great part but not the entire spectrum of nsLTPs sensitization, an eventual negativity to SPT performed using peach-LTP purified extract does not exclude a sensitization to a food other than peach.  Fresh foods used in PPTs is a mixture of allergenic and non-allergenic components, thus, a positivity does not ensure that specific LTP sensitization. | | | Being directed to allergenic and non-allergenic components of the suspected plant-food, sIgE do not ensure a certain diagnosis of nsLTPs sensitization.  A higher risk of anaphylaxis is related to patients polysensitized to 4 or more different nsLTPs. (19) | | Due to the potential risk, it must never be performed for foods already responsible of severe reactions. | |
|  | | | | | | | | | | |
| **Management** | | | | | | | | | | |
| **Education.** Explain what nsLTPs allergy is and the relevance of co-factors in favouring the outbreak of reactions, suggesting to avoid them. Provide general advice on foods that are more likely to provoke reactions, especially *Rosaceae* and nuts, even with information sheets. (31, 29) Provide a training for the use of epinephrine autoinjector. (44) Recommend the ingestion of fruit always peeled.  **Dietary elimination of the foods responsible for allergic reactions.** No need for dietary restriction in case of clinically irrelevant positivity to nsLTPs. (29)  **Prescription of antihistamines** for cutaneous symptoms and **glucocorticoids** for their eventual effect on protracted symptoms of anaphylaxis. (32)  **Prescription of the epinephrine auto-injector** in consideration of the unpredictable evolution of the allergy and of the role of eventual co-factors in favoring anaphylaxis, especially in case of previous anaphylaxis, young adulthood (because of more unpredictable behaviour), and long distance from healthcare. (32)  **Periodic follow up.** To assess the occurrence of allergy to new plant-foods and to refresh both educational recommendations and instructions about the use of epinephrine auto-injector. (31)  **Specific situations.** In case of FDEIA avoid physical exercise for the 4 hours subsequent the meal and pay particular attention to wheat. In case of FDNIH, when necessary, suggest the use of selective COX-2 inhibitors. Careful to hidden causes of allergens, as processed foods but also as cosmetic lotions. In case of contact urticaria of crop workers, suggest the use of gloves. In the case of Can s 3 allergy, avoiding of cannabis and its products.  **Allergen specific immunotherapy.** Not yet in routinely therapy, but a treatment option. (33) | | | | | | | | | | |

References

1. Asero, R. In patients with LTP syndrome food-specific IgE show a predictable hierarchical order. Eur Ann Allergy Clin Immunol. 2014 Jul;46(4):142-6. Erratum in: Eur Ann Allergy Clin Immunol. 2014 Nov;46(6):239.
2. Ebo, D.G., Swerts, S., Sabato, V., Hagendorens, M.M., Bridts, C.H., Jorens, P.G., et al. New food allergies in a European non-Mediterranean region: is Cannabis sativa to blame? Int Arch Allergy Immunol. 2013;161(3):220-8. doi: 10.1159/000346721.
3. Romano, A., Scala, E., Rumi, G., Gaeta, F., Caruso, C., Alonzi, C., et al. Lipid transfer proteins: the most frequent sensitizer in Italian subjects with food-dependent exercise-induced anaphylaxis. Clin Exp Allergy. 2012 Nov;42(11):1643-53. doi: 10.1111/cea.12011.
4. Dua, S., Ruiz-Garcia, M., Bond, S., Durham, S.R., Kimber, I., Mills, C., Roberts, G., Skypala, I., Wason, J., Ewan, P., Boyle, R., Clark, A. Effect of sleep deprivation and exercise on reaction threshold in adults with peanut allergy: A randomized controlled study. J Allergy Clin Immunol. 2019 Dec;144(6):1584-1594.e2. doi: 10.1016/j.jaci.2019.06.038.
5. Ruano-Zaragoza, M., Casas-Saucedo, R., De la Cruz Martinez, C.A., Araujo-Sanchez, G., Gelis, S., González, M.F., San Bartolomé, C., Pascal, M., Jiménez-Rodriguez, T.W., Gonzalez-Delgado, P., Fernandez-Sanchez, J., Muñoz-Cano, R., Bartra, J. Advances in the understanding of the cofactor effect in lipid transfer protein food allergy: From phenotype description to clinical management. Allergy. 2022 Jun;77(6):1924-1926. doi: 10.1111/all.15291.
6. Costanzo, G., Matolo, A., Saderi, L., Messina, M.R., Firinu, D., Barca, M.P., Serra, P., Corso, N., Sotgiu, G., Del Giacco, S. Cofactors, age at onset, allergic comorbidities and gender are different in patients sensitized to omega-5 gliadin and Pru p 3. Sci Rep. 2022 Dec 2;12(1):20868. doi: 10.1038/s41598-022-25368-y.
7. Tordesillas, L., Gómez-Casado, C., Garrido-Arandia, M., Murua-García, A., Palacín, A., Varela, J., et al. Transport of Pru p 3 across gastrointestinal epithelium - an essential step towards the induction of food allergy? Clin Exp Allergy. 2013 Dec;43(12):1374-83. doi: 10.1111/cea.12202.
8. Pastorello, E.A., Monza, M., Pravettoni, V., Longhi, R., Bonara, P., Scibilia, J., et al. Characterization of the T-cell epitopes of the major peach allergen Pru p 3. Int Arch Allergy Immunol. 2010;153(1):1-12. doi: 10.1159/000301573.
9. Asero, R. Peach-induced contact urticaria is associated with lipid transfer protein sensitization. Int Arch Allergy Immunol. 2011;154(4):345-8. doi: 10.1159/000321827.
10. Rihs, H.P., Armentia, A., Sander, I., Brüning, T., Raulf, M., Varga, R. IgE-binding properties of a recombinant lipid transfer protein from Cannabis sativa. Ann Allergy Asthma Immunol. 2014 Aug;113(2):233-4. doi: 10.1016/j.anai.2014.05.022.
11. Decuyper, I.I., Rihs, H.P., Van Gasse, A.L., Elst, J., De Puysseleyr, L., Faber, M.A., et al. Cannabis allergy: what the clinician needs to know in 2019. Expert Rev Clin Immunol. 2019 Jun;15(6):599-606. doi: 10.1080/1744666X.2019.1600403.
12. Faber, M.A., Van Gasse, A.L., Decuyper, I.I., Uyttebroek, A., Sabato, V., Hagendorens, M.M., et al. IgE-reactivity profiles to nonspecific lipid transfer proteins in a northwestern European country. J Allergy Clin Immunol. 2017 Feb;139(2):679-682.e5. doi: 10.1016/j.jaci.2016.06.016.
13. Skypala, I.J., Asero, R., Barber, D., Cecchi, L., Diaz Perales, A., Hoffmann-Sommergruber, K., et al.; European Academy of Allergy; Clinical Immunology (EAACI) Task Force: Non‐specific Lipid Transfer Protein Allergy Across Europe. Non-specific lipid-transfer proteins: Allergen structure and function, cross-reactivity, sensitization, and epidemiology. Clin Transl Allergy. 2021 May 18;11(3):e12010. doi: 10.1002/clt2.12010.
14. Asero, R., Pravettoni, V., Scala, E., Villalta, D. Lipid transfer protein allergy: A review of current controversies. Clin Exp Allergy. 2022 Feb;52(2):222-230. doi: 10.1111/cea.14049.
15. Borghesan, F., Mistrello, G., Roncarolo, D., Amato, S., Plebani, M., Asero R. Respiratory allergy to lipid transfer protein. Int Arch Allergy Immunol. 2008;147(2):161-5. doi: 10.1159/000137285.
16. Palacin, A., Quirce, S., Armentia, A., Fernández-Nieto, M., Pacios, L.F., Asensio, T., et al. Wheat lipid transfer protein is a major allergen associated with baker's asthma. J Allergy Clin Immunol. 2007 Nov;120(5):1132-8. doi: 10.1016/j.jaci.2007.07.008.
17. Lin, C.H., Li, L., Lyu, P.C., Chang, J.Y.. Distinct unfolding and refolding pathways of lipid transfer proteins LTP1 and LTP2. Protein J. 2004 Nov;23(8):553-66. doi: 10.1007/s10930-004-7882-2.
18. Scala, E., Till, S.J., Asero, R., Abeni, D., Guerra, E.C., Pirrotta, L., et al. Lipid transfer protein sensitization: reactivity profiles and clinical risk assessment in an Italian cohort. Allergy. 2015 Aug;70(8):933-43. doi: 10.1111/all.12635.
19. Ruano-Zaragoza, M., Somoza, M.L., Jiménez-Rodriguez, T.W., Soriano-Gomis, V., González-Delgado, P., Esteban-Rodriguez, A., et al. Lipid Transfer Protein Sensitization: Risk of Anaphylaxis and Molecular Sensitization Profile in Pru p 3-Sensitized Patients. Int Arch Allergy Immunol. 2021;182(5):425-432. doi: 10.1159/000511977.
20. Rizzi, A., Chini, R., Inchingolo, R., Carusi, V., Pandolfi, F., Gasbarrini, A., et al. Nickel allergy in lipid transfer protein sensitized patients: Prevalence and clinical features. Int J Immunopathol Pharmacol. 2020 Jan-Dec;34:2058738420974895. doi: 10.1177/2058738420974895.
21. Romano, A., Scala, E., Rumi, G., Gaeta, F., Caruso, C., Alonzi, C., et al. Lipid transfer proteins: the most frequent sensitizer in Italian subjects with food-dependent exercise-induced anaphylaxis. Clin Exp Allergy. 2012 Nov;42(11):1643-53. doi: 10.1111/cea.12011.
22. Niggemann, B., Beyer, K. Factors augmenting allergic reactions. Allergy. 2014 Dec;69(12):1582-7. doi: 10.1111/all.12532.
23. Sánchez-López, J., Araujo, G., Cardona, V., García-Moral, A., Casas-Saucedo, R., Guilarte, M., Torres, M.J., Doña, I., Picado, C., Pascal, M., Muñoz-Cano, R., Bartra, J. Food-dependent NSAID-induced hypersensitivity (FDNIH) reactions: Unraveling the clinical features and risk factors. Allergy. 2021 May;76(5):1480-1492. doi: 10.1111/all.14689.
24. Arena, A. Anaphylaxis to apple: is fasting a risk factor for LTP-allergic patients? Eur Ann Allergy Clin Immunol. 2010 Aug;42(4):155-8.
25. Wölbing, F., Fischer, J., Köberle, M., Kaesler, S., Biedermann, T. About the role and underlying mechanisms of cofactors in anaphylaxis. Allergy. 2013 Sep;68(9):1085-92. doi: 10.1111/all.12193.
26. de Silva, N.R., Dasanayake, W.M., Karunatilleke, C., Malavige, G.N. Food dependant exercise induced anaphylaxis a retrospective study from 2 allergy clinics in Colombo, Sri Lanka. Allergy Asthma Clin Immunol. 2015 Jul 25;11(1):22. doi: 10.1186/s13223-015-0089-6.
27. Asero, R., Pravettoni, V.. Anaphylaxis to plant-foods and pollen allergens in patients with lipid transfer protein syndrome. Curr Opin Allergy Clin Immunol. 2013 Aug;13(4):379-85. doi: 10.1097/ACI.0b013e32835f5b07.
28. Casas-Saucedo, R., de la Cruz, C., Araujo-Sánchez, G., Gelis, S., Jimenez, T., Riggioni, S., San Bartolomé, C., Pascal, M., Bartra Tomás, J., Muñoz-Cano, R. Risk Factors in Severe Anaphylaxis: Which Matters the Most, Food or Cofactors? J Investig Allergol Clin Immunol. 2022 Jul 22;32(4):282-290. doi: 10.18176/jiaci.0698.
29. Skypala, I.J., Bartra, J., Ebo, D.G., Antje Faber, M., Fernández-Rivas, M., Gomez, F., Luengo, O., Till, S.J., Asero, R., Barber, D., Cecchi, L., Diaz Perales, A., Hoffmann-Sommergruber, K., Pastorello, A.E., Swoboda, I., Konstantinopoulos, A.P., van Ree, R., Scala, E.; European Academy of Allergy & Clinical Immunology (EAACI) Task Force: Non-specific Lipid Transfer Protein Allergy Across Europe. The diagnosis and management of allergic reactions in patients sensitized to non-specific lipid transfer proteins. Allergy. 2021 Aug;76(8):2433-2446. doi: 10.1111/all.14797.
30. Decuyper, I.I., Rihs, H.P., Van Gasse, A.L., Elst, J., De Puysseleyr, L., Faber, M.A., et al. Cannabis allergy: what the clinician needs to know in 2019. Expert Rev Clin Immunol. 2019 Jun;15(6):599-606. doi: 10.1080/1744666X.2019.1600403.
31. Ridolo, E., Pucciarini, F., Kihlgren, P., Barone, A., Nicoletta, F., Peveri, S., et al. Lipid transfer protein syndrome: How to save a life through careful education. World Allergy Organ J. 2022 Sep 5;15(9):100683. doi: 10.1016/j.waojou.2022.100683.
32. Muraro A, Worm M, Alviani C, Cardona V, DunnGalvin A, Garvey LH, Riggioni C, de Silva D, Angier E, Arasi S, Bellou A, Beyer K, Bijlhout D, Bilò MB, Bindslev-Jensen C, Brockow K, Fernandez-Rivas M, Halken S, Jensen B, Khaleva E, Michaelis LJ, Oude Elberink HNG, Regent L, Sanchez A, Vlieg-Boerstra BJ, Roberts G; European Academy of Allergy and Clinical Immunology, Food Allergy, Anaphylaxis Guidelines Group. EAACI guidelines: Anaphylaxis (2021 update). Allergy. 2022 Feb;77(2):357-377. doi: 10.1111/all.15032. Epub 2021 Sep 1. PMID: 34343358.
33. García-Gutiérrez, I., Medellín, D.R., Noguerado-Mellado, B., Lillo Ordoñez, M.C., Abreu, M.G., Nogales, L.J., Rojas-Pérez-Ezquerra, P. Treatment with lipid transfer protein sublingual immunotherapy: slowing down new sensitizations. Asia Pac Allergy. 2021 Jan 25;11(1):e6. doi: 10.5415/apallergy.2021.11.e6.
